# Supplementary material for: Artificial light at night is a top predictor of bird migration stopover density
Source: Nat Commun. 2023 Dec 4;14:7446. doi: 10.1038/s41467-023-43046-z (PMC10696060; doi:10.1038/s41467-023-43046-z)
Supplement: Supplementary file 1 — Supplementary Information [file 41467_2023_43046_MOESM1_ESM.pdf]

Title: Artificial light at night is a top predictor of bird migration stopover density

Author List: Kyle G. Horton[1], Jeffrey J. Buler[2], Sharolyn J. Anderson[3], Carolyn S. Burt[1], Amy C. Collins[1]†, Adriaan M. Dokter[4], Fengyi Guo[5], Daniel Sheldon[6], Monika Anna Tomaszewska[7], Geoffrey M. Henebry[7,8]

Affiliations:
[1]Department of Fish, Wildlife, and Conservation Biology, Colorado State University, Fort Collins, Colorado, USA
[2]Department of Entomology and Wildlife Ecology, University of Delaware, Newark, Delaware, USA.
[3] Natural Sounds and Night Skies Division, National Park Service, 1201 Oakridge Dr., Suite 100, Fort Collins, CO, 80525, USA
[4]Cornell Lab of Ornithology, Cornell University, Ithaca, New York, USA
[5]Department of Ecology and Evolutionary Biology, Princeton University, Princeton, New Jersey, USA
[6]Manning College of Information and Computer Sciences, University of Massachusetts Amherst, Amherst, Massachusetts, USA
[7]Center for Global Change and Earth Observations, Michigan State University, East Lansing, Michigan, USA
[8]Department of Geography, Environment, and Spatial Sciences, Michigan State University, East Lansing, Michigan, USA
†Present address: Conservation Science Partners, Truckee, CA, USA

Correspondence and requests for materials should be addressed to K.G.H. **(**email: kyle.horton@colostate.edu**)**

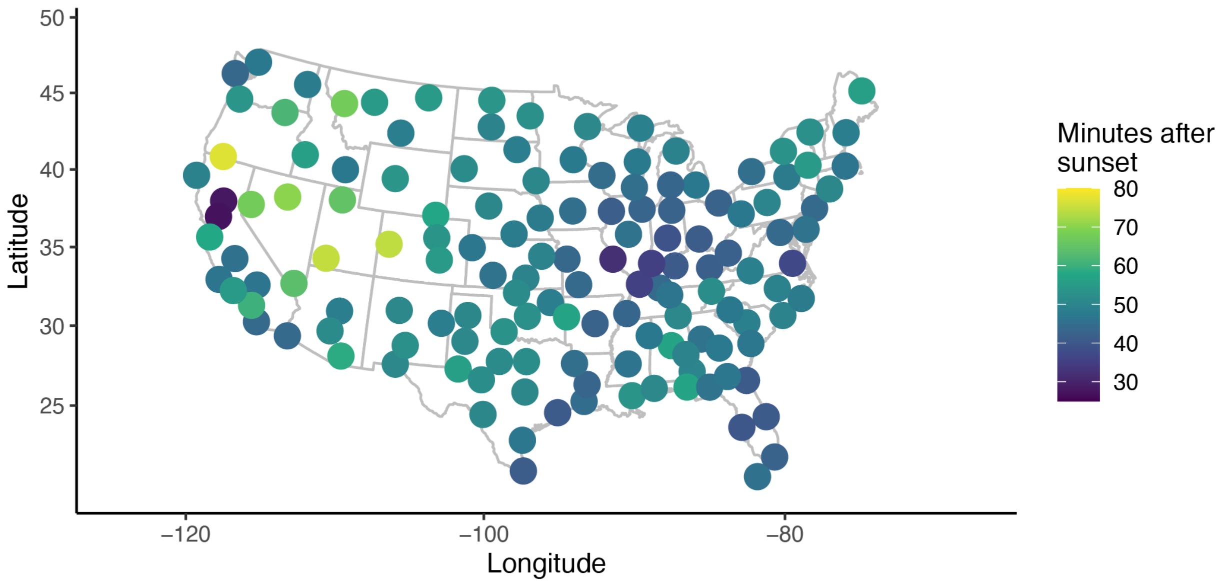

Fig. S1: Exodus sampling times (minutes after local sunset) for all 142 weather surveillance radar stations. Radar scans closest to these intervals were sampled to quantify nightly migrant exodus. These times were found to be the times that showed the greatest rate of change in reflectivity between sunset and 2.5 hours after sunset.

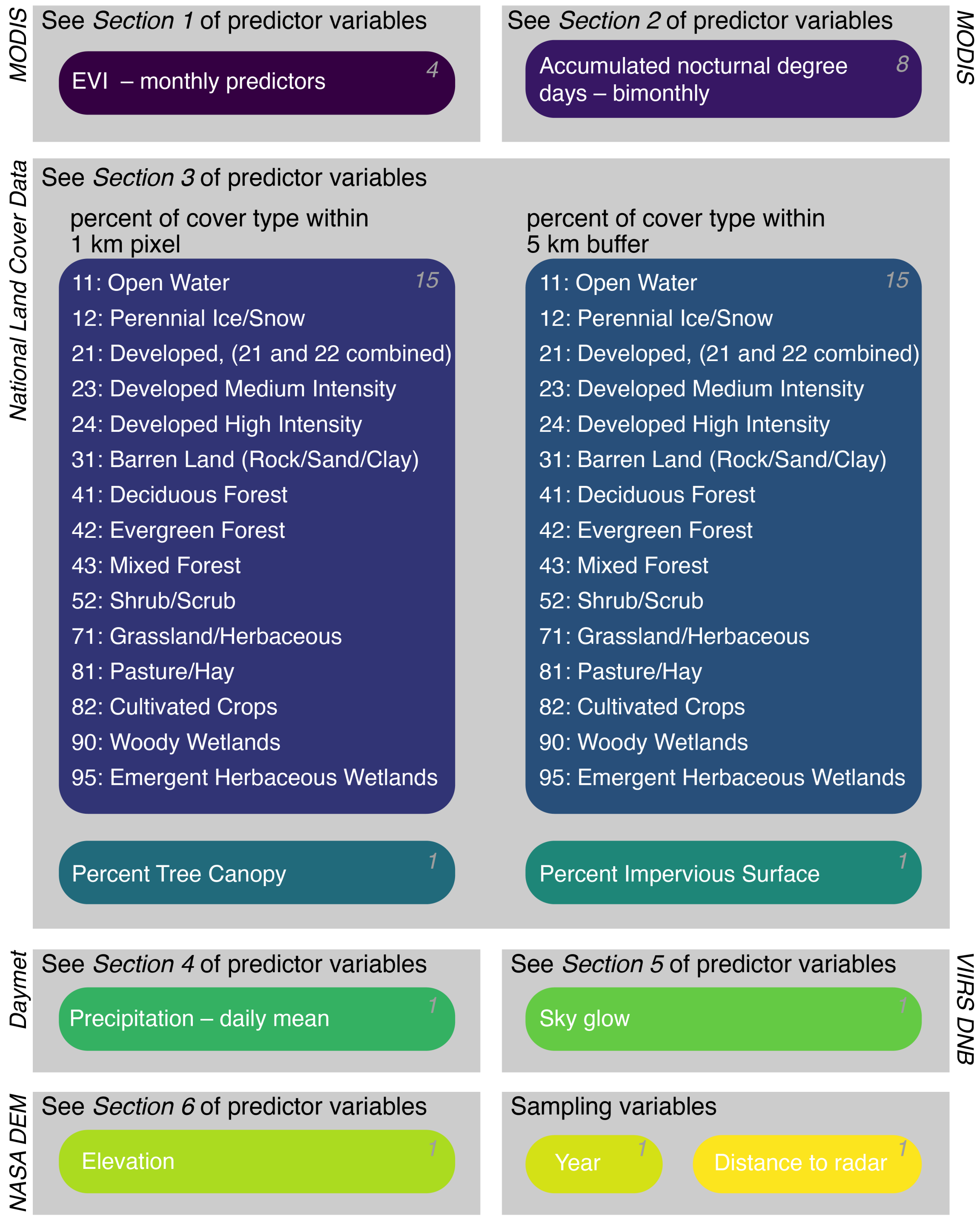

Fig. S2: Predictor variables assembled for niche modeling. Predictor variables by broad classes (e.g., Enhanced vegetation index (EVI), temperature, landcover, etc.). For each colored box, we denote the number of variables in each class in the top right corner — 49 predictors in total. For each gray box, we identify the source of the variables (e.g., MODIS, Nation Land Cover Data, etc.).

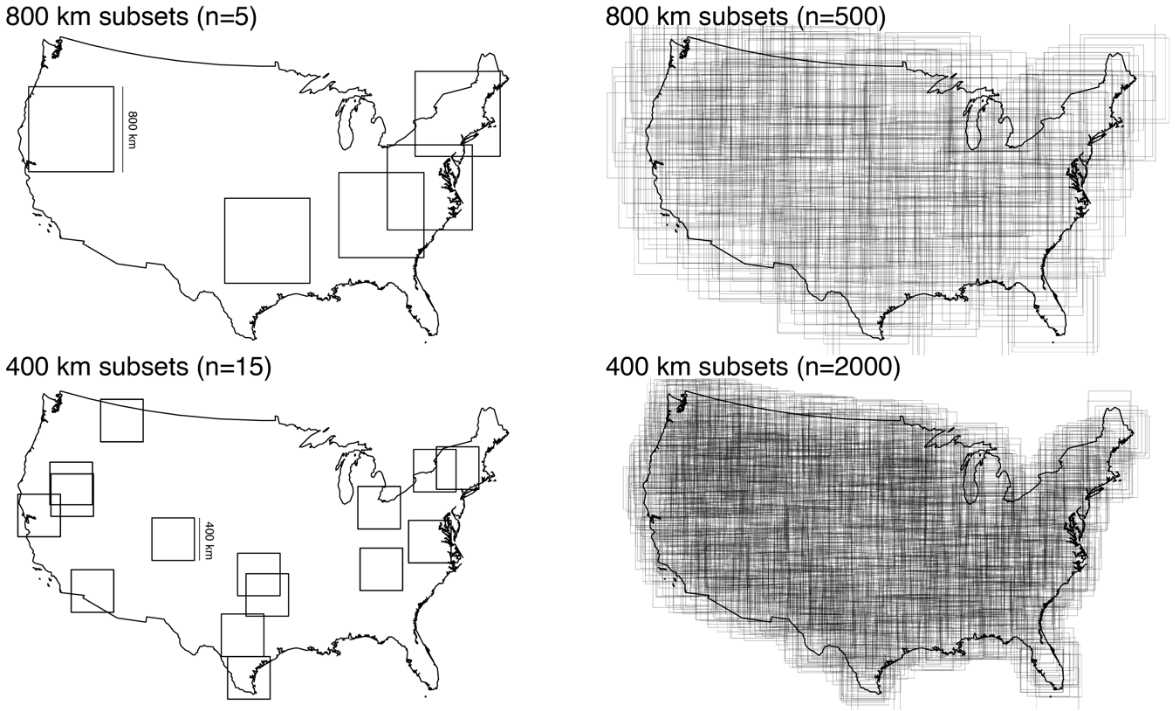

Fig. S3: Schematic of spatial subsets used in model training. In total, we assembled 2,500 unique models per season composed of 2,000 400-km subsets and 500 800-km subsets.

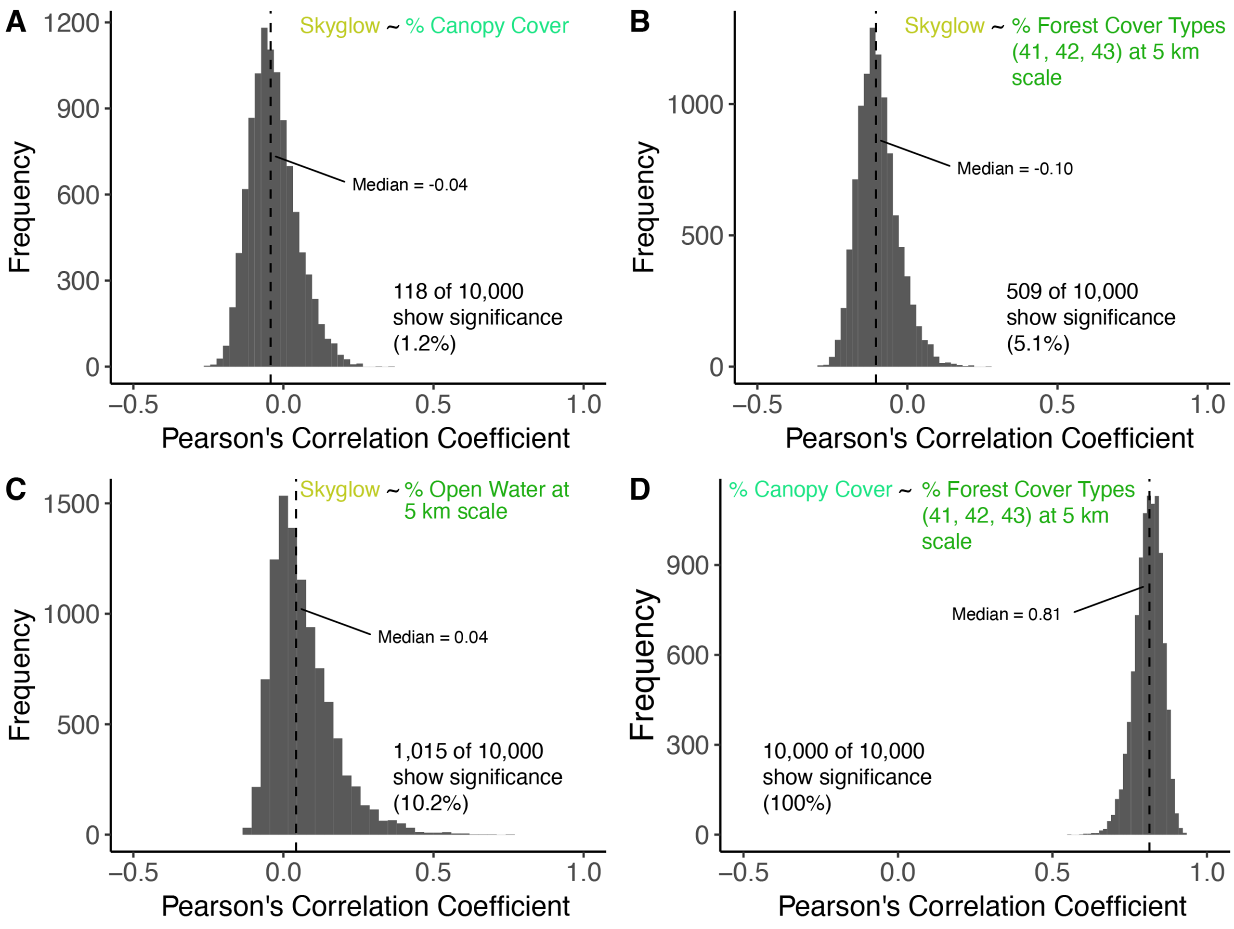

Figure S4: Pearson's correlation strength between model predictors, including (A) skyglow and % canopy cover, (B) skyglow and proportion of NLCD forest cover types (41, 42, and 43) within a 5 km buffer, (C) skyglow and proportion of NLCD open water within 5 km buffer, and (D) % canopy cover and proportion of NLCD forest cover types (41, 42, and 43) within a 5 km buffer. Correlations were conducted on 10,000 random selections of 100 locations from spring of 2016. Significance based on an alpha value of 0.05.

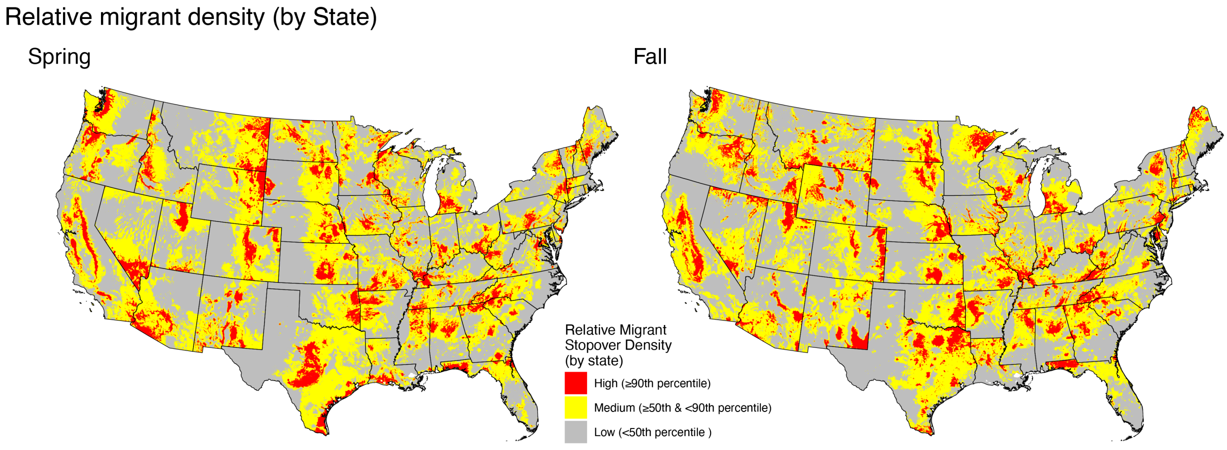

Fig. S5: Seasonal state-level relative hotspots. (A) Spring and (B) fall state-level relative stopover categories from predicted 2020 stopover density. For each state we identify three levels of relative migration stopover density (low, medium, high). Red shades (high) denote pixels above the 90th quantile of predicted stopover density, yellow (medium) pixels between the 50th and 90th quantile of migrant stopover density, and gray (low) showing pixels below the 50th quantile of migrant stover density.
