# Supplementary material for: Artificial light at night is a top predictor of bird migration stopover density
Source: Nat Commun. 2023 Dec 4;14:7446. doi: 10.1038/s41467-023-43046-z (PMC10696060; doi:10.1038/s41467-023-43046-z)
Supplement: Supplementary file 3 — Reporting Summary [file 41467_2023_43046_MOESM3_ESM.pdf]

nature portfolio

Corresponding author(s): Kyle G. Horton

Last updated by author(s): Oct 25, 2023

Reporting Summary

Nature Portfolio wishes to improve the reproducibility of the work that we publish. This form provides structure for consistency and transparency in reporting. For further information on Nature Portfolio policies, see our Editorial Policies and the Editorial Policy Checklist.

Statistics

For all statistical analyses, confirm that the following items are present in the figure legend, table legend, main text, or Methods section.

n/a	Confirmed	
☐	☒	The exact sample size (*n*) for each experimental group/condition, given as a discrete number and unit of measurement
☐	☒	A statement on whether measurements were taken from distinct samples or whether the same sample was measured repeatedly
☐	☒	The statistical test(s) used AND whether they are one- or two-sided<br>*Only common tests should be described solely by name; describe more complex techniques in the Methods section.*
☐	☒	A description of all covariates tested
☐	☒	A description of any assumptions or corrections, such as tests of normality and adjustment for multiple comparisons
☐	☒	A full description of the statistical parameters including central tendency (e.g. means) or other basic estimates (e.g. regression coefficient) AND variation (e.g. standard deviation) or associated estimates of uncertainty (e.g. confidence intervals)
☐	☒	For null hypothesis testing, the test statistic (e.g. F, t, r) with confidence intervals, effect sizes, degrees of freedom and P value noted<br>*Give P values as exact values whenever suitable.*
☒	☐	For Bayesian analysis, information on the choice of priors and Markov chain Monte Carlo settings
☒	☐	For hierarchical and complex designs, identification of the appropriate level for tests and full reporting of outcomes
☐	☒	Estimates of effect sizes (e.g. Cohen's *d*, Pearson's *r*), indicating how they were calculated

Our web collection on statistics for biologists contains articles on many of the points above.

Software and code

Policy information about availability of computer code

Data collection	No new data were collected for this analysis.
Data analysis	R Version 3.6.3

For manuscripts utilizing custom algorithms or software that are central to the research but not yet described in published literature, software must be made available to editors and reviewers. We strongly encourage code deposition in a community repository (e.g. GitHub). See the Nature Portfolio guidelines for submitting code & software for further information.

Data

Policy information about availability of data

All manuscripts must include a data availability statement. This statement should provide the following information, where applicable:

- Accession codes, unique identifiers, or web links for publicly available datasets
- A description of any restrictions on data availability
- For clinical datasets or third party data, please ensure that the statement adheres to our policy

We used open-access remote sensing data, including NOAA's NEXRAD products, Visible and Infrared Imaging Suite (VIIRS) Day Night Band (DNB) monthly cloud-free DNB composite products produced by the Earth Observation Group, Payne Institute for Public Policy, We used the 2016 and 2019 National Land Cover Database (NLCD) releases to characterize percent canopy cover (2016), percent impervious surface (2016, 2019), and land cover classification (2016, 2019), NASADEM data product (NASADEM_HGTv001) to capture elevation, Daymet (72) version 4 R1 to calculate mean daily precipitation for spring and fall, MODIS/Terra and MODIS/

Aqua Land Surface Temperature/Emissivity products (MOD11A2/MYD11A2 V061), and MODIS/Terra Vegetation Indices Monthly L3 Global product (MOD13A3 V061).

The weather surveillance radar and predictor data used in this study are available in the FigShare database under accession code 10.6084/m9.figshare.24438280.

Research involving human participants, their data, or biological material

Policy information about studies with human participants or human data. See also policy information about sex, gender (identity/presentation), and sexual orientation and race, ethnicity and racism.

Reporting on sex and gender	N/A
Reporting on race, ethnicity, or other socially relevant groupings	N/A
Population characteristics	N/A
Recruitment	N/A
Ethics oversight	N/A

Note that full information on the approval of the study protocol must also be provided in the manuscript.

Field-specific reporting

Please select the one below that is the best fit for your research. If you are not sure, read the appropriate sections before making your selection.

☐ Life sciences ☐ Behavioural & social sciences ☒ Ecological, evolutionary & environmental sciences

For a reference copy of the document with all sections, see nature.com/documents/nr-reporting-summary-flat.pdf

Ecological, evolutionary & environmental sciences study design

All studies must disclose on these points even when the disclosure is negative.

Study description	Leveraging over 10 million remote sensing observations, we developed seasonal contiguous US layers of bird migrant stopover density.
Research sample	We quantified migrant stopover density from reflectivity for 2016 to 2020 in spring (93 nights from March 15 to June 15) and fall (93 nights from August 15 to November 15). We downloaded all radar scans from sunset to 2.5 hours after local sunset from the Amazon Web Services repository (https://s3.amazonaws.com/noaa-nexrad-level2/index.html).
Sampling strategy	Using site-specific exodus sampling times, we selected and assembled all seasonal range-corrected and filtered scans closest to the period of interest. With scans assembled, we took the mean of stopover density across all sampling nights within a season-year interval. For each season-year combination, we mosaicked stopover densities from all 142 radar stations, taking the mean where overlap in sampling areas occurred. Lastly, we resampled to a 1 km resolution. These data served as our response variable for stopover modeling.
Data collection	All data used in the analysis were generated from remote sensing instruments. No new remote sensing data were collected, however we assembled and derived new layers from existing products.
Timing and spatial scale	We quantified migrant stopover density from reflectivity for 2016 to 2020 in spring (93 nights from March 15 to June 15) and fall (93 nights from August 15 to November 15). We quantified migration density in the contiguous United States.
Data exclusions	We removed data from weather contamination, clutter, and bat contamination. Precipitation was removed using the MISTNET algorithm, clutter from raster-based masks from regions with consistently high reflectivity, and bats from tailored screening for circular signatures of roost locations. For bats, we permanently removed these areas from further analysis (set to NA). In all, we screened 12 sites (KAMA, KCRP, KDFX, KEOX, KEWX, KGRK, KHGX, KLBB, KMXX, KSJT, KTLH, KVAX); states included Alabama, Florida, Georgia, and Texas. One site, KEWX (San Antonio, Texas) was so severely contaminated by bats that we removed the station completely from our analysis, reducing the number of radars to 142.
Reproducibility	All data were uploaded. 10.6084/m9.figshare.24438280.
Randomization	We sampled across the US to construct our niche models using 2000 randomly located 400km bounding boxes and 500 800km bounding boxes used for model training.
Blinding	N/A

Did the study involve field work? ☐ Yes ☒ No

Reporting for specific materials, systems and methods

We require information from authors about some types of materials, experimental systems and methods used in many studies. Here, indicate whether each material, system or method listed is relevant to your study. If you are not sure if a list item applies to your research, read the appropriate section before selecting a response.

Materials & experimental systems

n/a	Involved in the study
☒	☐ Antibodies
☒	☐ Eukaryotic cell lines
☒	☐ Palaeontology and archaeology
☒	☐ Animals and other organisms
☒	☐ Clinical data
☒	☐ Dual use research of concern
☒	☐ Plants

Methods

n/a	Involved in the study
☒	☐ ChIP-seq
☒	☐ Flow cytometry
☒	☐ MRI-based neuroimaging
